# Association of Serum Vitamin D level and COVID-19 infection: A Case-control Study

**DOI:** 10.5339/qmj.2022.48

**Published:** 2022-12-05

**Authors:** Najat Abdrabbo AlYafei, Bushra Naaz Fathima Jaleel, Abdel-Salam G. Abdel-Salam, Hamda Ali Al-Saadi, Samya Ahmad Al Abdulla

**Affiliations:** ^1^Primary Health Care Corporation, Qatar. Email & ORCID ID: nalyafei@phcc.gov.qa & https://orcid.org/0000-0002-8071-3646; ^2^Department of Mathmatics, Statistics and Physics, College of Art and Sciences, Qatar unitversity, Doha, Box. 2713, Qatar.

**Keywords:** COVID-19, SARS-CoV-2, RT-PCR, Vitamin D Deficiency, Serum 25(OH)D, Qatar

## Abstract

Background: Vitamin D is considered a potent modulator of the immune system, albeit its role in COVID-19 infection is a matter of debate. The present study aimed to estimate the association between serum vitamin D levels and COVID-19 among people in Qatar.

Methods: This case-control study, approved by the Institutional Review Board of Primary Health Care Corporation (PHCC) Qatar, retrospectively evaluated the principal public healthcare sector population data repository retrieved from the cloud-based Electronic Health Record (EHR) software-Cerner, during April 2020–2021. The health records of all adult patients aged >18 years who had undergone the reverse transcription-polymerase chain reaction (RT-PCR) test and whose medical records had documented serum 25-hydroxyvitamin D [25 (OH)D] levels were analyzed.

Results: A total of 924,173 EHRs were extracted, of which 62,451 EHR comprised of 16,446 (26.3%) COVID-19 patients and 46,005 (73.7%) negative-control group patients met the inclusion criteria. The odds ratio (OR) among different categories of vitamin D deficiency (VDD) revealed that people with mild/moderate VDD were 1.18 times (95% CI 1.126–1.258) and those with severe VDD were 1.90 times (95% CI 1.116–1.251) more likely to have COVID-19 infection when compared to the people with optimal serum vitamin D level. On applying multiple logistic regression, the odds of having COVID-19 infection were found to be 1.27 times (95% CI 1.184–1.371) higher among those with mild/moderate VDD and 1.32 times (95% CI 1.206–1.405) higher among those with severe VDD when compared to people with optimal vitamin D level (*p* < 0.001).

Conclusion: Our findings demonstrated a significant association between the suboptimal serum vitamin D level and COVID-19 infection. Further studies are required to determine the effects of VDD on the severity and outcomes of COVID-19 infections.

## Introduction

Since the time it was first identified in the Chinese city of Wuhan in December 2019 until date, the COVID-19 pandemic continues to present daunting challenges of immense magnitude to human survival, health, and well-being across the world. COVID-19 remains an enigma. Multiple variants of severe acute respiratory syndrome coronavirus 2 (SARS-CoV-2) have emerged and spread across the world, leading to the massive upheaval of fear, anxiety, stress, and depression among the general population and especially among healthcare workers.^1–3^ This international public health crisis of substantial mortality has accelerated research to elicit its association with various conditions/diseases and to explore avenues for its prevention.

Several studies have reported the potential of micronutrients like zinc, selenium, and iron in modulating the immune system and their deficiency correlating with COVID-19 infection.^4,5^ Vitamin D is considered a potent modulator of the immune system. Therefore, several researchers examined the plausible association between vitamin D deficiency (VDD) and COVID-19 infections.^6–9^ However, the evidence was inconsistent and inconclusive as some studies indicated an association but others did not.^10–13^ This finding may be attributed to variations in methodology, small samples, confounding factors, or diverse healthcare systems. Hence, it is imperative to accumulate evidence and better understand the role of VDD in the COVID-19 pandemic. The prevalence rate of VDD among people in Qatar is exceptionally high, reaching 71.4%.^14^ Owing to the extensive skin coverage, scarce exposure to natural sunlight, and longer in-door dwelling to avoid the extreme soaring heat and arid climate most of the year, it is of considerable interest to determine the association of serum vitamin D levels with COVID-19 infection in this country. It is hypothesized that VDD is found frequently among people testing positive for COVID-19 infection. Therefore, this retrospective study was undertaken to estimate the association between serum vitamin D levels and COVID-19 among people who undertook reverse transcription-polymerase chain reaction (RT-PCR) test for COVID-19 infection during the study period of April 2020–2021.

## Methods

### Study setting

Qatar is a peninsula state, with an area of 11,521 km^2^, located amid the western coast of the Arabian Gulf. The population of Qatar at the end of April 2021 was 2,646,854 people (72% males and 28% females)^15,16^. Qatari nationals account for approximately < 15% of the total population, with the remaining being comprised of expatriates.^17^ The first positive COVID-19 patient was reported in Qatar on February 29, 2020.^18^

This case-control study retrospectively evaluated the principal public healthcare sector population data repository of the Primary Health Care Corporation (PHCC) and Hamad Medical Corporation (HMC) retrieved from the cloud-based Electronic Health Record (EHR) software—Cerner. PHCC delivers primary healthcare services through 27 health centers strategically located in 3 main health regions—northern, central, and western, covering the entire State of Qatar.^19^ The HMC operates 12 hospitals (9 specialist hospitals and 3 community hospitals) and provides specialist facilities and services for secondary and tertiary care in the country.^20^

Health records of all adult patients aged >18 years who had undergone RT-PCR test and whose medical records, including the serum 25-hydroxyvitamin D [25 (OH)D] levels, were recorded in the EHR were analyzed (n = 62,451). Vitamin D assessment was considered valid if it was conducted within a year before the date of RT-PCR testing. Patients whose serum vitamin D level was assessed within 14 days of the RT-PCR test for COVID-19 were excluded, as this duration coincided with the incubation period for coronavirus. Patients who had received any form of vitamin D supplementation (tablets/injection) after serum 25 (OH) D testing and before the RT-PCR test for COVID-19 were excluded from the study.

This study was approved by the Institutional Review Board of Primary Health Care Corporation, State of Qatar (Reference no: PHCC/DCR/2021/05/034). The data analysis was performed without the use of any identifiable data. However, demographic characteristics, serum vitamin D level, COVID-19 infection status, smoking status, body mass index (BMI), and the medical history of comorbidities/medication related to COVID-19 and vitamin D metabolism were extracted by the Business and Health Intelligence team of PHCC.

The assessed cases included patients with positive testing on RT-PCR for COVID-19, based on the national guidelines, whereas those with negative RT-PCR test results constituted the control group. Both COVID-19 patients and the control subjects were representatives of the population in Qatar during April 2020–2021 time frame. RT-PCR test was performed using nasopharyngeal and/or oropharyngeal swabs and was considered positive based on the quantitative detection of ribonucleic acid from the SARS-CoV-2 virus at the Department of Laboratory Medicine and Pathology, HMC.

The serum vitamin D level assessment reports were retrieved from EHS. The Serum 25 (OH)D levels were categorized as follows: severe VDD (serum 25 (OH)D < 10 ng/mL), mild/moderate VDD category (10–20 ng/mL), and optimal serum vitamin D (>20 ng/mL).^14^

### Statistical analysis

Data cleaning involved the elimination of the population with invalid serum vitamin D reports and checking for dates of COVID-19 testing within the specified study time range. Data analysis was performed on SPSS 28 (SPSS Inc., Chicago, IL, USA).

The continuous variables were presented as the means and standard deviations, while frequencies and percentages were used to express the categorical variables. The Chi-Square test was performed to assess the association between categorical variables, and the Mann–Whitney U-test was performed to compare the means between COVID-19 patients and control subjects. The significance level was set at 5%. The logistic regression model accounted for the confounding variables and shortlisted the most significant variables. All variables were initially entered into the model. Subsequently, significant variables were further analyzed in another model, and the adjusted odds ratio (OR) was calculated. Finally, the models were evaluated using the Omnibus test, Cox Snell R^2^, Negelkerke R^2^, and Hosmer–Lemeshow goodness-of-fit tests.

## Results

**Figure 1.**
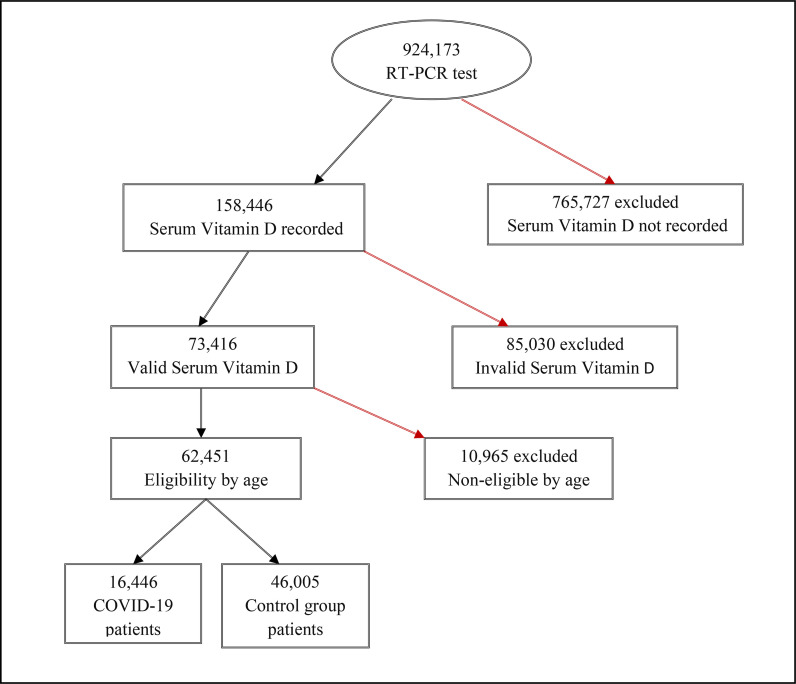
Flow diagram depicting the process of sample selection

A total of 924,173 EHRs were extracted, which comprised the potentially eligible population who had undergone RT-PCR testing for COVID-19 during April 2020–2021. Among these, 62,451 had valid serum 25(OH) D assessment documented in the EHR and were of age >18 years. Hence, the final study sample consisted of 16,446 (26.3%) COVID-19 patients who were RT-PCR-positive and 46,005 (73.7%) control subjects (Figure 1).

**Figure 2.**
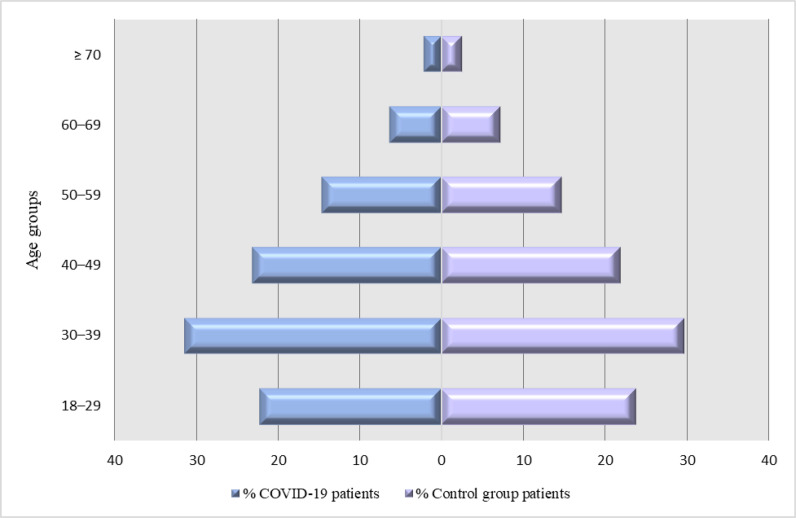
Age distribution of the COVID-19 patients and the control subjects

The general characteristics of the study population are shown in Table 1. The mean age of the COVID-19 patients and the control subjects were 41.10 ± 13.07 and 41.19 ± 13.61 years, respectively (*p* = 0.48). When the study population was stratified by age, a statistically significant difference (p < 0.001) was detected between the groups, with more people in the 30–39 and 40–49 year-age groups who tested RT-PCR positive. Age distribution of the COVID-19 patients and the control subjects are depicted in Figure 2 (*p* < 0.001).

A statistically significant variation in COVID-19 patients was also observed by nationality. Lower disease prevalence was detected among Qataris (30.7%) when compared to non-Qataris (69.3%) (*p* < 0.001). The mean BMI among COVID-19 patients was 30.24 ± 7.32 kg/m^2^, which was significantly marginally higher than that of the control subjects (29.64 ± 8.14 kg/m^2^) (*p* < 0.001). A greater proportion of people in the COVID-19 group (46.3%) were obese relative to the control group (41.1%) (*p* < 0.001). Gender distribution and smoking status showed no statistically significant difference between the two groups.

Table 2 presents the study population's comorbidities and medication usage data. Analysis revealed a statistically significant difference between the COVID-19 patients and the control subjects with regard to some of the clinical characteristics. COVID-19 patients were more likely to have diabetes, hypertension, chronic obstructive pulmonary disease (COPD), respiratory diseases, kidney disease, and chronic corticosteroid therapy.

Table 3 depicts the distribution of VDD among the study population, based on the most recent serum 25 (OH) D measurement. We recorded that 38.3% of COVID-19 patients had optimal serum vitamin D levels, 49% had mild/moderate VDD, and 12.7% had severe VDD. A statistically significant difference was recorded with respect to the level of serum vitamin D between the two groups. Univariate logistic regression analysis was employed to assess the association between VDD and COVID-19 infection. The OR among the different categories of VDD revealed that people with mild/moderate VDD were 1.18 times (95% CI 1.126–1.258) more likely and people with severe VDD were 1.90 times (95% CI 1.116–1.251) more likely to have COVID-19 infection when compared to people with optimal serum vitamin D level. Furthermore, when the effect of seasonal variations of vitamin D was considered, it was found that people who had their most recent serum vitamin D level assessed in the winter season were more likely to have COVID-19 infection (26.3%) when compared to people who had the assessment done in the summer season (25.2%) (*p* < 0.001).

The multiple logistic regression model was initially built by entering all 14 variables. After controlling the effect of other significant variables, the final logistic regression model demonstrated an independent association between the serum vitamin D level and COVID-19 infection. The odds of having COVID-19 infection were found to be 1.27 times (95% CI 1.184–1.371) higher among those with mild/moderate VDD and 1.32 times (95% CI 1.206–1.405) higher among those with severe VDD when compared to people with optimal vitamin D level (*p* < 0.001) (Table 4). Testing positive for COVID-19 was also associated with age < 50 years, obesity, non-Qatari nationality, vitamin D assessment in the winter season, diabetes, COPD/respiratory diseases, and chronic corticosteroid therapy. Together with VDD, the predictive accuracy of this logistic regression model was 73.4%.

## Discussion

This study is one of the few, large-scale, retrospective case-control studies to date to investigate the role of vitamin D in COVID-19 infection. Multivariate analysis of our data suggested a statistically significant association between the suboptimal serum vitamin D level and COVID-19 infection after controlling for the significant confounding variables. Hence, suboptimal serum vitamin D level was independently associated with COVID-19 infection.

The vital role of vitamin D in the regulation of calcium and phosphorus metabolism within the human body and its deficiency impacting the musculoskeletal system is indisputable.^21,22^ Another well-recognized role of vitamin D is immunomodulation, which encompasses its critical influence on innate and adaptive immunities.^23^ The putative role of vitamin D in the immune response against viral infections such as influenza, dengue, hepatitis, and HIV has been extensively researched.^24–27^ Amid the ongoing COVID-19 pandemic, the contributory influence of VDD has drawn great interest and driven numerous scientific publications to add to the existing body of knowledge.

It has been ascribed that the contributory role of vitamin D in COVID-19 infection is pleotropic. First, vitamin D supports the production of antimicrobial peptides in the respiratory epithelium by monocytes and neutrophils.^28^ It also promotes the degradation of the SARS-CoV-2 virus through endolysosomes.^29^ VDD weakens the innate immune response and enables higher SARS-CoV 2 load, which results in the overactivation of the adaptive immune system with increased release of cytokines such as TNFα and IL-1β, leading to the production of a cytokine storm and progression in the severity of the infection and increased mortality.^30^ VDD also causes the overactivation of the pulmonary renin-angiotensin system (RAS), leading to respiratory syndrome. RAS is partly regulated by angiotensin-converting enzyme 2 (ACE2), which also acts as a primary receptor for SARS-CoV-2 entry into the cells. Hence, VDD can exacerbate COVID-19 through its effects on ACE2.^31^

Previous systematic reviews and meta-analyses have clearly indicated an association between vitamin D levels and COVID-19 infection. Teshome et al.^32^ concluded that individuals with an insufficient level of vitamin D were 80% more likely to acquire COVID-19 infection when compared to those with a normal level of vitamin D (OR = 1.80; 95%CI: 1.72–1.88). Another meta-analysis of 23 studies including 11901 patients by Ghasemian et al.^33^ suggested the odds of getting infected with SARS-CoV-2 to be 3.3 times higher among individuals with VDD (95% CI: 2.5–4.3) and the chance of developing severe COVID-19 to be approximately 5 times higher in patients with VDD (OR: 5.1, 95% CI: 2.6–10.3).

Similar to our findings, Meltzer et al. conducted multivariate analysis among 489 patients and reported a relative risk of testing positive for COVID-19 to be 1.77 higher among patients with VDD status.^7^ Another study among 156 COVID-19 patients and 204 controls in India revealed a significant association between vitamin D levels and COVID-19 infection with an adjusted OR of 3.295 (1.25–8.685).^34^ A study from Wuhan^35^ revealed, through multiple logistic regression, that VDD is associated with COVID-19 infection.

Abdollahi et al. from Iran, Ye et al. from China, Hernández et al. from Spain, Carpagnano et al. from Italy, Angelidi et al., from the USA, and Diaz-Curiel et al. from Spain also reported significantly higher rates of VDD in COVID-19 patients.^36–41^

Conversely, a study on 348,598 UK Biobank participants did not find any significant association between VDD and testing positive for COVID-19 infection.^42^ This difference may be attributed to the limitation in the analytical approach applied as they took into account the serum 25 (OH) D levels of 10–14 years before the COVID-19 pandemic and were not contemporaneous.

Our study's strength lies in the investigation of the important contribution of seasonal variations in vitamin D levels on COVID-19 infections. We found that individuals with serum vitamin D levels assessed during winter were more likely to have COVID-19 infection. These findings agree with those of Qu et al.^43^ who investigated the roles of environmental factors in the spread and transmission of COVID-19 infections. Qu et al.^43^ found that respiratory tract infections were more frequent in the winter seasons, particularly in the northern latitudes, when compared to the summer season, with high infections of COVID-19 reported during the winter months. Our study revealed evidence that VDD is a significant contributor to COVID-19 infection, especially in the winter.

We acknowledge that the results of this study were based on patient data from the principal public healthcare sector of Qatar and may not be nationally representative as the semi-government, private, and voluntary health institutions were not included.

## Conclusion

Our results revealed a strong and significant association between the suboptimal serum vitamin D level and COVID-19 infection after controlling for the confounders in the regression model. The VDD status, as indicated by low 25(OH)D levels, strongly indicated a contribution to the development or onset of COVID-19 infection. Hence, daily vitamin D supplementation may be recommended as the potential strategy to prevent COVID-19, particularly for vulnerable adults, obese adults, and individuals with comorbidities. Further studies are warranted to determine the effects of VDD on the severity and outcomes of COVID-19 infections.

### Conflicts of Interest

The authors declare no conflicts of interest.

### Authors’ Contribution

All authors contributed to the design, data collection, data analysis, and interpretation of the results. NAA, BNFJ, HAA and SAA led the data collection. ASG led the data analysis. NAA, BNFJ, ASG, SAA and HAA led the interpretation and presentation of results. NAA and BNFJ wrote the initial draft of the manuscript. All the authors read and approved the final manuscript.

## Figures and Tables

**Table 1:** General characteristics of the study population

	COVID-19 patients (n = 16446)	Control group patients (n = 46005)	p-value

Age, Mean ± SD	41.10 ± 13.07	41.19 ± 13.61	0.486

Age categories			

18–29	3647 (22.2 %)	10941 (23.8 %)	

30–39	5159 (31.4 %)	13676 (29.7 %)	

40–49	3801 (23.1 %)	10082 (21.9 %)	

50–59	2415 (14.7 %)	6759 (14.7 %)	< 0.001

60–69	1058 (6.4 %)	3336 (7.3 %)	

≥ 70	366 (2.2 %)	1211 (2.6 %)	

Gender			

Male	6586 (40%)	18436 (40.1%)	

Female	9860 (60%)	27569 (59.9)	0.956

Nationality			

Qatari	5042 (30.7%)	15326 (33.3%)	

Non-Qatari	11404 (69.3%)	30679 (66.7%)	< 0.001

BMI, Mean ± SD	30.24 ± 7.32	29.64 ± 8.14	< 0.001

BMI Categories			

Underweight ≤ 18.5	127 (1.3%)	473 (1.7%)	

Healthy ≥ 18.5 ≤ 25	1889 (19.1%)	6120 (22.5%)	

Overweight ≥ 25 ≤ 30	3296 (33.3%)	9449 (34.7%)	< 0.001

Obese ≥ 30	4581 (46.3%)	11201 (41.1%)	

Smoking Status			

Smoker	74 (0.4%)	203 (0.4%)	0.470

Non-smoker	16372 (99.6%)	45802 (99.6%)	


**Table 2:** Clinical characteristics of the study population

	COVID-19 patients	Control group patients	p-value

Diabetes			

Present	4623 (28.1%)	11486 (25%)	< 0.001

Absent	11823 (71.9%)	34519 (75%)	

Hypertension			

Present	4253 (25.9%)	11200 (24.3%)	< 0.001

Absent	12193 (74.1%)	34805 (75.7%)	

CVD			

Present	622 (3.8%)	1803 (3.9%)	0.224

Absent	15824 (96.2%)	44202 (96.1%)	

COPD/Respiratory disease			

Present	1862 (11.3%)	4757 (10.3%)	< 0.001

Absent	14584 (88.7%)	41248 (89.7%)	

Chronic liver disease			

Present	130 (0.8%)	368 (0.8%)	0.474

Absent	16316 (99.2%)	45637 (99.2%)	

Chronic kidney disease			

Present	360 (2.2%)	885 (1.9%)	< 0.05

Absent	16086 (97.8%)	45120 (98.1%)	

Chronic Corticosteroid therapy			

Present	1291 (7.8%)	2512 (5.5%)	< 0.001

Absent	15155 (92.2%)	43493 (94.5%)	


**Table 3:** Serum vitamin D level and seasonality of vitamin D assessment

	COVID-19 patients	Control group patients	p-value	Crude OR (95%CI)

Serum Vitamin D categories				

Optimal	6290 (26.7%)	17259 (73.3%)		1.18 (1.126–1.258)

Mild/ Moderate	8067 (26.9%)	21974 (73.1%)	< 0.001	1.90 (1.116–1.251)

VDD	2089 (23.6%)	6772 (76.4%)		

Severe VDD				

Seasonality of Serum Vitamin D assessment				

Summer	8986 (25.2%)	26605 (74.8%)	< 0.001	

Winter	7460 (26.3%)	19400 (72.2%)		


**Table 4:** Multivariate logistic regression analysis

								95% C.I. for EXP(B)	

		B	S.E.	Wald	df	Sig.	Exp(B)	Lower	Upper

Step 8h	Age_Categorical (1)	-.201	.032	40.465	1	< .001	.818	.769	.870

	BMI_Categorical (1)	.193	.024	63.545	1	< .001	1.213	1.157	1.272

	Serum Vit- D level			50.012	2	< .001			

	Serum Vit- D level (1)	.242	.037	42.265	1	< .001	1.274	1.184	1.371

	Serum Vit- D level (2)	.264	.039	45.967	1	< .001	1.302	1.206	1.405

	Nationality (1)	-.063	.025	6.440	1	.011	.939	.895	.986

	Seasonality Vit D analysis (1)	.108	.024	20.749	1	< .001	1.114	1.064	1.167

	Diabetes (1)	.238	.030	63.694	1	< .001	1.268	1.196	1.345

	COPD/Respiratory diseases (1)	.078	.036	4.736	1	.030	1.081	1.008	1.161

	Chronic corticosteroid therapy (1)	.395	.043	85.804	1	< .001	1.484	1.365	1.613

	Constant	-1.396	.038	1383.735	1	< .001	.248		


h. Variable(s) entered: Age_Categorical (>50 years), BMI_Categorical (obese), Serum Vit- D level (1- Mild/ moderate VDD Vs. optimal: 2- Severe VDD Vs. Optimal), Nationality (Qatari), Seasonality of Vit D analysis (Winter), Diabetes, COPD/ Respiratory diseases, Chronic corticosteroid therapy. Omnibus test: p < 0.001, Cox & Snell R Square = 0.009, Nagelkerke R Square = 0.013, Hosmer and Lemeshow Test p = 0.210, Method: Backward LR. B = regression coefficient, SE = standard error, Wald = Wald Chi-Square test, df = Degrees of freedom, Exp (B) = Exponential value of B or Adjusted Odds Ratio, 95 % CI = 95 % confidence interval.
